# The Effect of Plasma Treatment on the Mechanical Properties of HDPE/Bamboo Fiber Composites

**DOI:** 10.3390/polym17070983

**Published:** 2025-04-04

**Authors:** Zihan Ma, Yan Wu, Hongyan Wang, Jian Zhang, Shaofei Yuan

**Affiliations:** 1 Bamboo Research Institute of Zhejiang Academy of Forestry, Hangzhou 310023, China; 19852852008@163.com (Z.M.); zhjianzj@126.com (J.Z.); 2 College of Furnishings and Industrial Design, Nanjing Forestry University, Nanjing 210037, China; wuyan@njfu.edu.cn

**Keywords:** plasma treatment, high-density polyethylene (HDPE), bamboo, surface wettability, HDPE/bamboo composite materials

## Abstract

In order to improve the interface compatibility of HDPE/bamboo fiber composites, O_2_ and N_2_ plasma were used to treat the HDPE surface, the effects of plasma treatment power, time, and different flow rates on HDPE surface wettability were analyzed, the optimal process of plasma to improve HDPE surface wettability was determined, and a mathematical model between HDPE surface energy and the plasma treatment process was established. The effect of plasma treatment on the mechanical properties of HDPE/bamboo fiber composites was further studied, and the surface morphology and surface chemical structure of the materials before and after plasma treatment were analyzed. The results show that the surface wettability of HDPE can be significantly improved after O_2_ and N_2_ plasma treatment, which can further enhance the interface compatibility of HDPE/bamboo fiber composites and improve the impact and tensile strength of the composites (impact strength increased by 19.91% and 19.55% after O_2_ and N_2_ plasma treatment, respectively; tensile strength increased by 16.47% and 12.48%, respectively). The optimal process parameters for enhancing the interface compatibility of the two plasma composites are 1100 W, 13 s, and 1.75 L/min (O_2_ plasma), and 1100 W, 13 s, 2.5 L/min (N_2_ plasma); between the two, N_2_ plasma has a better effect on the surface wettability of HDPE than O_2_ plasma treatment.

## 1. Introduction

High-density polyethylene (HDPE) is widely used in polymer material processing owing to its high hardness, tensile strength, creep resistance, abrasion resistance, electrical insulation, and ease of processing [1,2]. However, the hydrophobicity of HDPE surfaces causes poor surface adhesion strength when combined with polar materials, leading to low compatibility [3,4]. Surface modification via plasma treatment, which was developed in the 1990s, has the advantages of simplicity, high efficiency, and no pollution. It can be used to treat material surfaces at low temperatures, avoiding damage to the internal structure and overall performance of the material while changing its surface properties [5]. The plasma surface modification of HDPE to enhance the wettability of HDPE surfaces has been studied previously. Yao Yaoguang et al. studied the effect of plasma treatment with different gases on the surface energy of HDPE and found that the surface energy of HDPE decreased and its surface energy and adhesion increased greatly after plasma treatment. The order of influence of the four plasma gases on the surface energy of HDPE was Ar > H_2_ > O_2_ > N_2_ = air [5]. In this study, L-CN was prepared by calcining B-CN, and RF-L-CN was obtained through N_2_ plasma modification of L-CN. It was found that the thin layer structure inhibited the recombination of photogenerated charges in C_3_N_4_. Plasma modification did not alter the material’s microstructure, crystal structure, framework unit structure, or chemical functional group structure, but it increased the nitrogen content in L-CN, further promoting the separation of photogenerated charges in C3N4. As a result, RF-L-CN/SPE exhibited a strong photocurrent signal in PBS electrolyte containing AA [6].

Arpagaus et al. studied the effect of plasma treatment on the surface wettability of HDPE; their results indicated that –C = –O– and –COOH functional groups were added to the surface of HDPE. This caused the water contact angle to decrease, the surface tension to increase, and the surface wettability to increase [7]. To enhance biocompatibility, Mora-Cortes et al. designed a low-pressure radiofrequency plasma technique that facilitates the formation of primary amine (-NH_2_) groups on PET films using diethylenetriamine (DTA). Additionally, fluorine-containing functional groups play a crucial role in the chemical grafting process, and the resulting grafted polar groups improve wettability [8]. Although traditional surface modification methods such as corona discharge treatment provide hydrophilicity, it is currently difficult to achieve long-lasting hydrophilic surfaces. Therefore, Yoshihisa et al. investigated the modification process of plastics using microwave plasma to obtain durable hydrophilic surfaces. They developed a two-step treatment process using argon plasma followed by argon/oxygen plasma. The results showed that the two-step plasma treatment technique maintained the hydrophilicity of the plastic surface for at least 80 days [9]. Yao Yaoguang et al. investigated the degree of surface crosslinking of high-density polyethylene (HDPE) under various plasma treatment conditions. The performance of plasma modification was studied through surface energy and adhesive bonding strength measurements. The experiments revealed that the surface crosslinking of HDPE occurs during plasma treatment, and the surface energy and adhesion of the material are enhanced after plasma treatment [5].

Although plasma treatment can improve the surface wettability of HDPE and enhance its surface bonding performance, the relationship between the modification effects and plasma treatment parameters (power, time, and gas flow rate) is unclear. In this study, O_2_ plasma and N_2_ plasma were used to treat HDPE surfaces, and the effects of the treatment power, time, and gas flow rate on surface wettability were investigated. Furthermore, response surface optimization was employed to optimize the plasma treatment process and establish an innovative response surface model between the surface energy and treatment parameters, thus providing theoretical support for plasma treatment improving the wettability of HDPE surfaces in industrial applications. The modified HDPE particles and bamboo powder were prepared by blending injection molding with different bamboo powder particle sizes, different ratios, and different temperatures, and the optimal process was determined, the effect of plasma treatment on the physical and mechanical properties of HDPE/bamboo fiber composites was further studied, and the surface morphology and surface chemical structure of the materials before and after plasma treatment were analyzed.

## 2. Materials and Methods

### 2.1. Materials and Instruments

HDPE, purchased from Dongguan Sun Tat Yuen Insulation Materials Co., Ltd.,Dongguan, China, was in the form of a recycled pellet, which was melted into sheets with a thickness of 1.5 mm and was then cut into specimens with dimensions of 40 × 40 × 1.5 mm^3^. Before the experiment, the specimens were soaked in anhydrous ethanol for ultrasonic cleaning to remove surface impurities and pollutants. After cleaning, the HDPE specimens were dried at room temperature and placed in a sealable bag. Diiodomethane (CH_2_I_2_), purity > 99.0%, was obtained from Shanghai Maclin Biochemical Technology Co., Ltd., Shanghai, China. Anhydrous ethanol, purity > 99.9%, was obtained from Chengdu Colon Chemical Co., Ltd., Chengdu, China. Distilled water (H_2_O) was prepared in the laboratory. Oxygen (O_2_) and nitrogen (N_2_), purity > 99.9%, were obtained from Shanghai Yunguang Industrial Gas Co., Ltd., Shanghai, China. The moso bamboo powder was purchsed from Hunan Weixing Bamboo and Wood Products Co., Ltd., Loudi, China, which particle sizes were divided into 40, 60, and 80 mesh with a moisture content of 10% to 12%.

The following equipment was used in this research: a cold-temperature plasma instrument (PG-3000K, Nanjing Suman Plasma Technology Co., Ltd., Nanjing, China), optical contact angle measurement instrument (DSA100, Germany KRÜSS Co., Ltd., Hamburg, Germany), ultrasonic cleaner (SB25-12, Ningbo Xinzhi Biotechnology Co., Ltd., Ningbo, China), Fourier-transform infrared spectrometer (Scientific Nicolet iS10, Thermo Fisher Scientific, Waltham, MA, USA), X-ray diffractometer (X’Pert Pro, PANalytical B.V., Almelo, The Netherlands), scanning electron microscope (SEM, S-3400N, Hitachi, Tokyo, Japan), microsampler (tip, 250 µL, Shanghai Gaoge Industry and Trade Co., Ltd., Shanghai, China), and Universal learning testing machine (model CMT4202 Shenzhen Sansi Zongheng Technology Co., Ltd., Shenzhen, China).

### 2.2. Plasma Treatment of HDPE Surfaces

**Figure 1:**
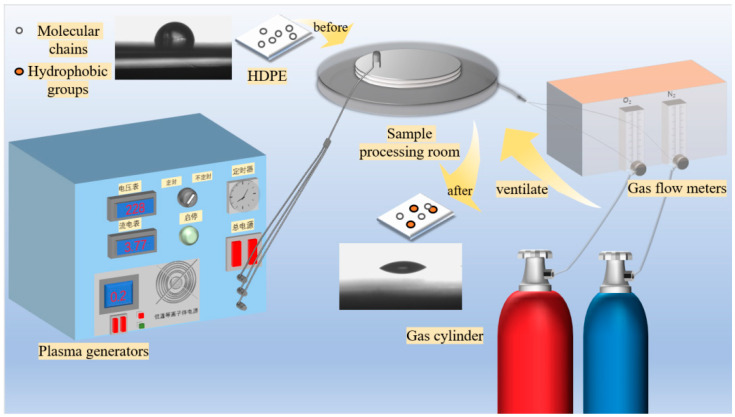
Schematic diagram of plasma treatment equipment.

With O_2_ and N_2_ used as working gases, a three-factor, three-level experimental method was employed to investigate the effects of treatment gas, power, and time on the wettability of HDPE surfaces. Plasma treatment: The prepared HDPE specimens were placed in the center of the sample treatment chamber in low-temperature plasma modification equipment, as shown in Figure 1. The gas flow meter was opened, and either O_2_ or N_2_ was introduced. The gas flow was adjusted to the desired rate, and the equipment was activated to generate plasma. After a certain duration, the equipment was turned off to complete HDPE specimen treatment. The treated specimens were removed and stored in self-sealing bags for later use. The selection of plasma parameters was based on previous studies. During plasma treatment, the treatment power was set to 800 W, 1000 W, and 1200 W; the treatment time was set to 5, 10, and 15 s; and the gas flow rate was set to 0.5, 1.5, and 2.5 L/min.

### 2.3. HDPE/Bamboo Fiber Composite Material Production

Before treatment, the bamboo fibers and HDPE were dried in an oven at 50 °C for 24 h, and then removed and stored in sealed bags for later use. Subsequently, the bamboo fibers and HDPE were mixed in batches according to the orthogonal experimental design and fed into a twin-screw extruder with a screw speed of 20 rpm. The mixing temperature was set to 160, 180, and 200 °C; after mixing for 10 min, the mixture was extruded. The extruded material was crushed in a grinder to form fine bamboo/plastic composite particles after cooling, which were then fed into a micro-injection molding machine. The molding process utilized the principle of thermoplastic melt flow at high temperatures. The holding pressure time was set to 10 s, the temperature of the injection mold was 40 °C, and the barrel temperature was 190 °C.

**Figure 2:**
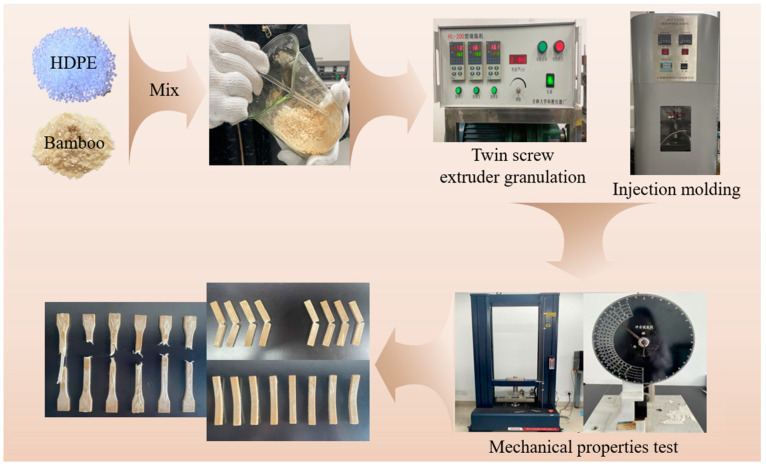
Preparation process of bamboo/plastic composite materials.

Figure 2 is the process of bamboo/plastic composite materials.

### 2.4. Surface Wettability Testing and Characterization

The HDPE surface was characterized by dynamic contact angle measurements using the sessile drop method. H_2_O and CH_2_I_2_ were used as test liquids, and the angle formed between the droplet and the surface at the initial contact point was considered the contact angle. Six points were tested on each sample, the average of which was taken as the test result. Furthermore, the surface energy was calculated using the Young–Good–Girifalco–Fowkes equation [10,11].

### 2.5. Optimization of Plasma Treatment Parameters and Establishment of Surface Energy Curve Models for Response Surface Method

In this study, the power, time, and gas flow rate of plasma treatment were each varied among three levels according to the response surface method. The surface energy was measured as the response value. Multiple regression equations between the surface energy and the treatment power, time, and gas flow rate were obtained using Design-Expert 11.0 to fit the experimental results. The experimental results were analyzed using variance analysis.

### 2.6. Orthogonal Experimental Design for HDPE/Bamboo Fiber Composites

Orthogonal experimental design is a method used to study multi-factor and multi-level designs. It employs the principle of orthogonality to design orthogonal tables, selecting representative points from full factorial experiments for testing.

The mechanical testing of bamboo/plastic composite materials was conducted using an orthogonal experimental design with three factors and three levels, based on the selected equipment temperature, bamboo powder particle size, and bamboo powder/HDPE raw material ratio. The optimal preparation conditions were derived through comprehensive analysis using SPSS Statistics 27, Excel, and Design-Expert 11.0. In this context, A represents temperature; B represents particle size; C represents the ratio; and D represents a blank test. The data of schemes with the same number of levels were summed to obtain K1, K2, and K3. Dividing them by 3 yielded the average calculation results for three levels, denoted as k1, k2, and k3. The difference between the maximum and minimum values for each level is the range R. The orthogonal experimental design is shown in Table 1.

### 2.7. Testing of Mechanical Properties of HDPE/Bamboo Fiber Composites

The impact strength was measured with reference to the national standard GB/T 1043.2-2018 [12], the notch was 8 mm, the pendulum impact method was adopted, the pendulum energy was 2 J, and the average value of 6 measurements was taken.

The bending strength was tested with reference to GB/T 9341-2008 [13] (three-point bending), the tensile rate was 5 mm/min, the size of the specimen was 600.0 mm × 7.0 mm × 4.0 mm, and the average value of 6 measurements was taken.

The tensile strength was tested with reference to GB/T 1040.1-2006 [14], the size of the specimen was 150.0 mm × 10.0 mm × 4.0 mm, and the average value of 6 measurements was taken.

### 2.8. Surface Microstructure Analysis

The surface microstructure before and after the modification of bamboo/plastic composites was observed using a scanning electron microscope (SEM) with an accelerating voltage of 12.5 kV. The samples were dried, cleaned, and gold-coated before testing.

### 2.9. X-Ray Diffraction Analysis

The powder of bamboo/plastic composites before and after modification was tested using X-ray diffraction analysis. The testing angle ranged from 10° to 40° at a scanning speed of 2°/min. The changes in the crystallinity of cellulose and the composites were analyzed.

### 2.10. FTIR Testing

The chemical group changes in the sample powder were analyzed using FTIR in the wavenumber range of 400–4000 cm^−1^. The presence or absence of functional groups before and after the modification of bamboo/plastic composites and the formation of new functional groups were analyzed.

### 2.11. XPS Testing

The excitation source was a monochromatic aluminum Kα source, the energy analyzer had a fixed pass energy of 100 eV, the spot size was 650 μm, and the scanning range was 0–1400 eV.

### 2.12. Thermogravimetric Analysis

The sample mass ranged from 8 to 10 mg. To fully characterize the pyrolysis properties of the sample in an inert atmosphere, high-purity nitrogen gas (purity ≥ 99.99%) was used, with the nitrogen flow rate maintained at 20 mL/min during testing. The thermogravimetric analyzer heated the sample from 30 °C to 800 °C at a rate of 10 °C/min. Sample mass was below 10 mg.

## 3. Results and Discussion

### 3.1. Plasma Treatment Improves Surface Wettability of HDPE

Table 1 and Table 2 show the results of O_2_ and N_2_ plasma treatments on the surface energy of HDPE, respectively. Based on the data on contact angles and surface energy from Table 3 and Table 4, it can be observed that after N_2_ plasma treatment, the surface energy of HDPE is higher. N_2_ plasma treatment has a more obvious improvement effect on the surface wettability of HDPE compared with O_2_ plasma treatment.

The experimental results were subjected to multivariate regression fitting using Design-Expert 11.0, and the multivariate regression equations between the surface energy of HDPE after O_2_ plasma and N_2_ plasma treatment, treatment power, treatment time, and gas flow rate were obtained.

The regression equation for O_2_ plasma is

Y = 57.59 + 1.55A + 1.19B + 0.0806C − 1.14AB − 0.3170AC − 1.44BC − 0.9750A^2^ − 1.36B^2^ − 0.6441C^2^

and the regression equation for N_2_ plasma is

Y = 0.4320A + 2.07B + 3.63C + 0.5270AB + 0.1137AC − 0.7625BC − 0.1325A^2^ − 0.6198B^2^ + 2.99C^2^

In the regression equations, Y, A, B, and C represent the surface energy, treatment power, treatment time, and gas flow rate, respectively.

### 3.2. Variance Analysis of the Models

Table 3, Table 4, Table 5 and Table 6 present the variance results of the quadratic model and the fit statistics of the effects of O_2_ and N_2_ plasma treatments on the surface energy of HDPE. The respective F-values of the two regression models were 4.85 and 22.31. The *p*-value for O_2_ plasma treatment was less than 0.0001, indicating that the difference in regression mode was very significant, and that for N_2_ plasma treatment was less than 0.1, indicating that the difference in regression mode was significant. The *p*-values for the lack of fit were 0.2343 and 0.1483; both were greater than 0.05, indicating that the model differences were not significant. The lack-of-fit tests for the regression equation show no significance, indicating that the lack of fit caused by errors is not significant. Therefore, these equations are reliable [15].

The regression coefficients (R_2_) were 0.8617 and 0.9663 for O_2_ and N_2_, respectively. Both values were greater than 85%, indicating that the equations have a good degree of fit. The regression equation can be used instead of actual test values to describe the relationship between the variables and response value [16,17,18]. The correction coefficients (adjusted R_2_) were 0.6840 and 0.9230, respectively, indicating that the model can explain 68.4% and 99.05% of the results for the O_2_ and N_2_ plasma treatments, respectively. The data in the table show that the design of the experiment was reliable and the error was small; therefore, the models are suitable for analyzing and predicting the actual surface energy of HDPE after O_2_ and N_2_ plasma treatment. The coefficients of variation were 2.08 and 1.60, respectively, which are low, indicating the high accuracy of the mathematical model and that this result can be used in prediction analysis.

Combined with the data in the table above, the degree of influence of each factor can be obtained by comparing the F-values. In this experiment, the degree of influence of the surface energy of HDPE after O_2_ plasma was in the order of treatment power > treatment time > gas flow rate, and that after N_2_ plasma was in the order of gas flow rate > treatment time > treatment power.

### 3.3. Response Surface Optimization: 3D Graph Analysis

**Figure 3:**
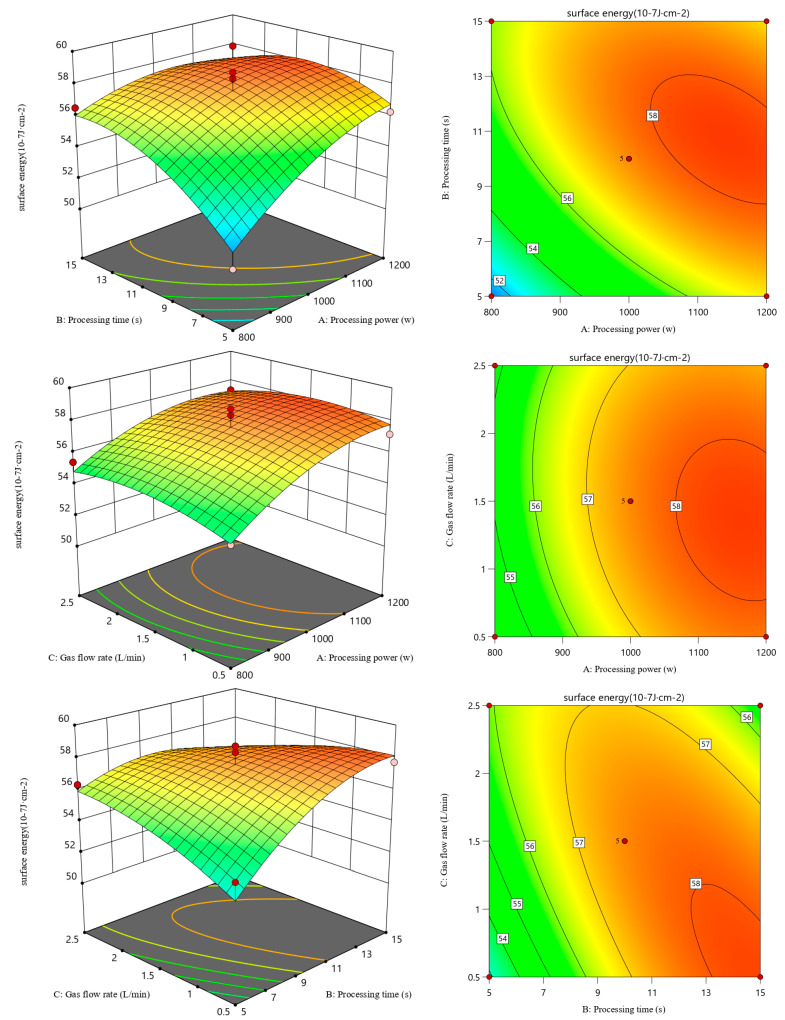
Response surface and contour of surface energy of HDPE after O_2_ plasma treatment.

Figure 3 shows the response surface and contour of the surface energy of HDPE under the interaction of various O_2_ plasma treatment conditions. The response surface diagram directly reflects the degree of influence of a given independent variable on the dependent variable. The larger the slope of the fitted surface and the denser the contours in the response surface diagram, the more significant the influence of the factor on the degree of correlation [19]. When the contour line is elliptical, the interaction is significant. Conversely, when the contour line is circular, there is no interaction between the two variables [20,21]. In Figure 3, it can be seen that the slope of the fitted surface of the influence of the treatment power and treatment time of O_2_ plasma on the surface energy of HDPE is steep, indicating a significant interaction between treatment power and treatment time [22]. The interaction between O_2_ plasma treatment power and time has the greatest effect on the surface energy of HDPE, while the interaction between time and gas flow rate has the least effect. From the contour plot, it can be inferred that higher treatment power results in higher surface energy.

**Figure 4:**
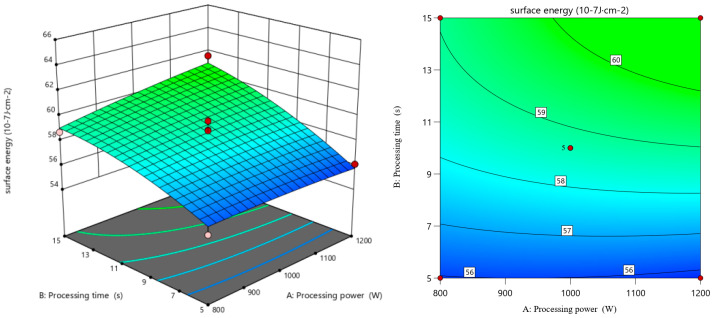

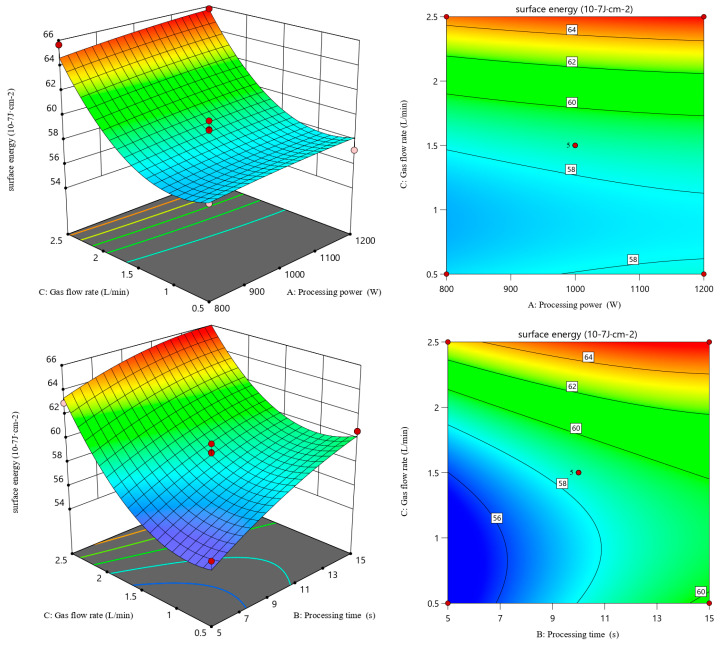
Response surface and contour of surface energy of HDPE after N_2_ plasma treatment.

Figure 4 shows the response surface and contour of the surface energy of HDPE under the interaction of various N_2_ plasma treatment conditions. It can be seen that the interaction between N_2_ plasma treatment gas flow rate and time has the greatest effect on the surface energy of HDPE, and the interaction between power and time has the least effect. Studies have shown that there is a significant increase in variation in the surface energy of HDPE with increasing treatment time and gas flow rate [23]. The same result is obtained in this experiment.

### 3.4. Validation Tests

Design-Expert 11.0 was used to solve the regression fitting equations for O_2_ and N_2_ plasma treatment, and the optimal manufacturing conditions were determined. For O_2_ plasma treatment, the optimal treatment power, time, and gas flow rate were 1114.037 W, 12.971 s, and 1.757 L/min, respectively, resulting in a predicted surface energy of 58.358 × 10^−7^ J·cm^−2^. For N_2_ plasma treatment, the respective optimal parameters were 1081.188 W, 12.585 s, and 2.481 L/min, resulting in a predicted surface energy of 65.945 × 10^−7^ J·cm^−2^. It can be inferred that under optimal processing conditions, the surface energy of HDPE modified by N_2_ plasma was greater than that modified by O_2_ plasma. Based on practical production requirements, the optimized conditions were adjusted for verification. For both O_2_ and N_2_ plasma treatments, the optimized treatment power was 1000 W and the optimized treatment time was 10 s. The optimized gas flow rate for O_2_ was 1.5 L/min, and that for N_2_ was 2.5 L/min. Validation experiments were performed under these conditions, and the surface energy results were 58.33 × 10^−7^ J·cm^−2^ for O_2_ plasma treatment and 60.51 × 10^−7^ J·cm^−2^ for N_2_ plasma treatment. There is little error compared to the model’s predicted results, which shows that the optimal process parameters obtained through the response surface method are accurate and reliable and, thus, have application value.

### 3.5. SEM Analysis

**Figure 5:**
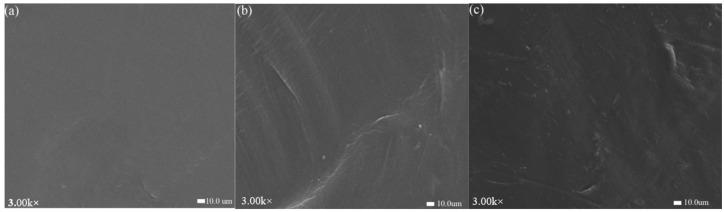
SEM images of HDPE surface before and after plasma treatment: (**a**) untreated, (**b**) O_2_ plasma-treated, and (**c**) N_2_ plasma-treated.

SEM was employed to observe the changes in HDPE surface morphology before and after optimal plasma treatment; the magnification for the images is 3000×. The SEM results are shown in Figure 5. The untreated HDPE surface appeared relatively smooth, whereas after plasma treatment, distinct etch marks, small white spots, and grooves were evident on the surface, accompanied by a significant increase in surface roughness. Some studies indicated that after plasma treatment, there were obvious plasma etch marks, with uneven potholes formed by cold plasma roughening the surface of the material [24]. The main cause of surface roughening during plasma treatment may be the bombardment of high-energy particles from cold plasma on the plastic surface, which etch and thereby alter the physical structure of the surface, leading to increased roughness [25]. The results of this experiment are consistent with those of previous studies. As shown in Figure 4, the degree of etching caused by O_2_ and N_2_ plasma treatments varies, and N_2_ plasma treatment results in higher surface roughness, which is consistent with the surface energy and response surface results.

### 3.6. X-Ray Photoelectron Spectroscopy (XPS)

**Figure 6:**
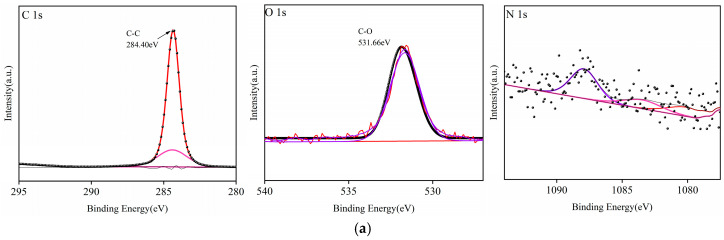

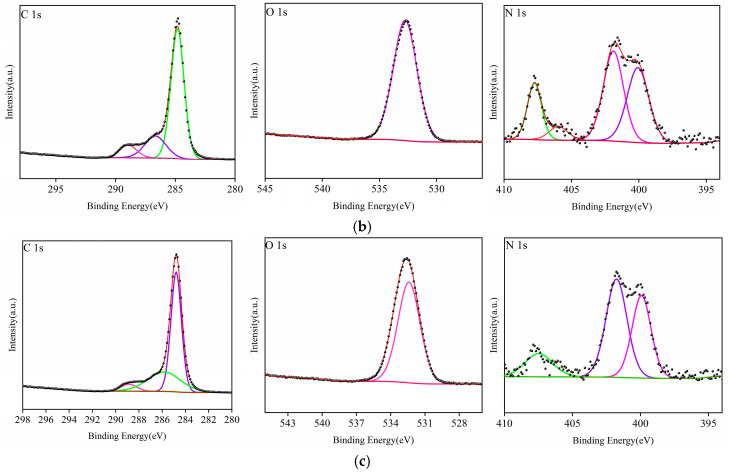
C1s, N1s, and O1s peak spectra of XPS on HDPE surface before and after plasma treatment: (**a**) untreated, (**b**) O_2_ plasma-treated, and (**c**) N_2_ plasma-treated.

The XPS results are shown in Figure 6 and Table 7. It can be seen that the C1s spectrum of the untreated HDPE surface is symmetric with a binding energy of 284.4 eV and is composed mainly of C–C/C–H bonds (285.0 eV) [26]. After plasma treatment, the major elements on the HDPE surface remain unchanged, primarily consisting of oxygen, carbon, and nitrogen. The C1s peak exhibits significant asymmetry with a tail toward higher energies, indicating the introduction of oxygen-containing functional groups on the surface of HDPE after O_2_ and N_2_ plasma treatment. Through waveform identification, these oxygen-containing functional groups were determined to primarily comprise two types, –C = O– and –O = C–O–, which is consistent with previous studies [27,28,29]. The wettability of a polymer surface is closely related to its functional groups. The introduction of oxygen or nitrogen polar groups on the surface can generate specific forces in the vertical direction at the interface. These forces are synergistically generated by hydrogen bonds and intermolecular forces between various polar groups, leading to an overall improvement in polymer wettability [30,31,32]. In the N1s peak spectra, it can be seen that the peak on the HDPE surface after N_2_ plasma treatment is significantly higher than that before treatment, with this difference possibly arising from the formation of amino groups owing to the introduction of –C–NH_2_, C = N, and other groups [33,34]. However, further analysis is required to determine the specific cause. The introduction of these polar groups significantly enhanced the polarity of the HDPE surface, which in turn significantly reduced the contact angle between the treated sample and water, increased the surface energy, and improved the hydrophilicity.

### 3.7. Mechanical Properties of HDPE/Bamboo Fiber Composites

#### 3.7.1. The Effects of Different Ratios, Temperatures, and Particle Sizes of Bamboo Powder on Mechanical Properties of HDPE/Bamboo Fiber Composites

**Figure 7:**
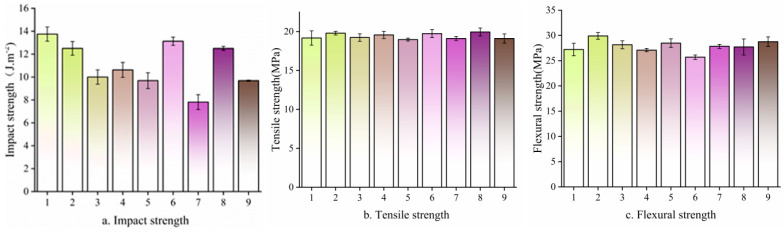
Mechanical properties of HDPE/bamboo fiber composites.

The ratio of bamboo fiber to plastic, the particle size of bamboo fiber, and the blending temperature have a significant impact on the mechanical properties of composite materials. The lower the content of bamboo fiber, the more uniform the distribution of bamboo fiber in composite materials. The larger the particle size of bamboo fiber, the better the reinforcement effect of bamboo fiber. The higher the blending temperature, the better the melting performance and mechanical properties of bamboo/plastic composites. In order to achieve good mechanical properties (including impact, tensile, and bending strength), we studied the effects of the bamboo fiber and HDPE ratio, bamboo fiber particle size, and melting temperature on the properties of composites. Figure 7 shows the effects of nine different ratios and the temperature and particle size of bamboo powder on the mechanical properties of HDPE/bamboo fiber composites. As can be seen in Figure 7a, differences in formulation have the greatest impact on the change in impact strength, and the optimal process parameters for impact strength are 1:9 (bamboo and HDPE ratio), 160 °C (temperature), and 60 mesh (particle size of bamboo). As can be seen in Figure 7b, differences in the ratios and the temperature and particle size of bamboo powder have the least influence on tensile properties, and the optimal process parameters are 2:8, a temperature of 180°, and a particle size of 80 mesh. Figure 7c shows that the optimal process parameters for bending performance are 1:9, a temperature of 180°, and a particle size of 80 mesh. From this mechanical property analysis, it can be seen that the optimal process parameters of bamboo/plastic composites are as follows: the ratio is 1:9, the temperature is 180°, and the particle size is 80 mesh.

#### 3.7.2. Mechanical Properties of Plasma-Modified HDPE/Bamboo Fiber Composites

**Figure 8:**
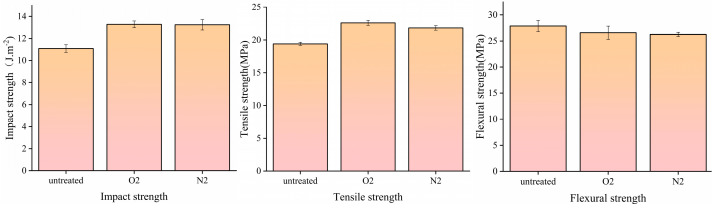
Mechanical properties of plasma-modified HDPE/bamboo fiber composites.

The results for the mechanical properties of the HDPE/bamboo fiber composites are shown in Figure 8, which mainly presents the impact strength, tensile, and flexural properties. In Figure 8, the flexural strength of plasma-modified HDPE/bamboo fiber composites decreases compared to the untreated ones, with O_2_ plasma and N_2_ plasma reducing it by 4.61% and 5.76%, which is due to the fact that bamboo fibers were used as rigid fillers, and their addition can inevitably lead to an increase in the rigidity and decrease in the flexibility of the HDPE/bamboo fiber composites. In contrast to the flexural strength, the impact strength increased by 19.91% and 19.55% after O_2_ and N_2_ plasma treatment, respectively; the tensile strength increased by 16.47% and 12.48%, respectively. At the same time, as polar fillers, bamboo fibers can form an interfacial phase separation morphology with non-polar HDPE, which leads to a decrease in the continuity of the HDPE matrix and limits its performance [35,36]. With the addition of bamboo fibers, their agglomeration in the plastic matrix intensifies, which can easily cause the internal stress concentration of the composite material and increase the probability of defects. The HDPE/bamboo fiber composites are composed of hydrophilic bamboo fibers and a hydrophobic HDPE matrix. Prior to modification, the poor interfacial compatibility between these two components led to a decrease in the mechanical properties.

Plasma treatment improved the hydrophilicity of the HDPE surface and enhanced interfacial bonding with the bamboo fibers. As a result, the impact strength and tensile strength of the modified HDPE/bamboo fiber composites were improved, indicating that surface wettability and interfacial adhesion were enhanced after plasma modification. However, this improvement in the impact strength and tensile strength of the modified HDPE/bamboo fiber composites also indicates that the surface ability became stronger after plasma modification, and the interface bonding between bamboo fiber and the HDPE matrix was enhanced. The unmodified HDPE/bamboo fiber composites had only simple mechanical winding and the interaction force was weak, and the agglomeration of bamboo fiber will cause stress concentration when the composites are subjected to external forces, resulting in a decrease in flexural strength [37,38,39].

### 3.8. Microscopic Morphology Analysis of HDPE/Bamboo Fiber Composites

**Figure 9:**
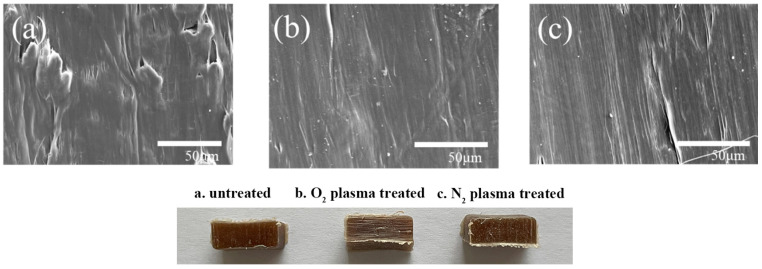
Microscopic morphology of HDPE/bamboo fiber composites before and after modification: (**a**) untreated, (**b**) O_2_ plasma-treated, and (**c**) N_2_ plasma-treated.

Figure 9 shows the morphology of the cross-sectional surface before and after the modification of the optimal HDPE/bamboo fiber composites; the magnification for the images is 600×. In Figure 9a, there are obvious voids and holes in the cross-section, because in the process of melt blending, bamboo fiber and HDPE only form a simple mechanical bond, and the internal structure is heterogeneous, so the voids and pores not only reduce the interfacial bonding strength but are also more easily damaged when stressed [40,41]. In Figure 9b,c, it can be clearly seen that the distribution of bamboo fiber and HDPE is more uniform, the combination is tighter, the surface has become smoother, the gap is reduced, the fiber distribution is more uniform, the strength is better, the wrapping is better, and the mechanical properties of the HDPE/bamboo fiber composites are increased. After comparison, it can be seen that the fusion degree of O_2_ plasma modification was better than that of N_2_ plasma, with the cross-sectional surface demonstrating that the fusion of HDPE/bamboo fiber composites modified by O_2_ plasma was better, and the bamboo fiber could be evenly dispersed in the HDPE plastic matrix.

### 3.9. XRD Analysis of HDPE/Bamboo Fiber Composites Before and After Modification

**Figure 10:**
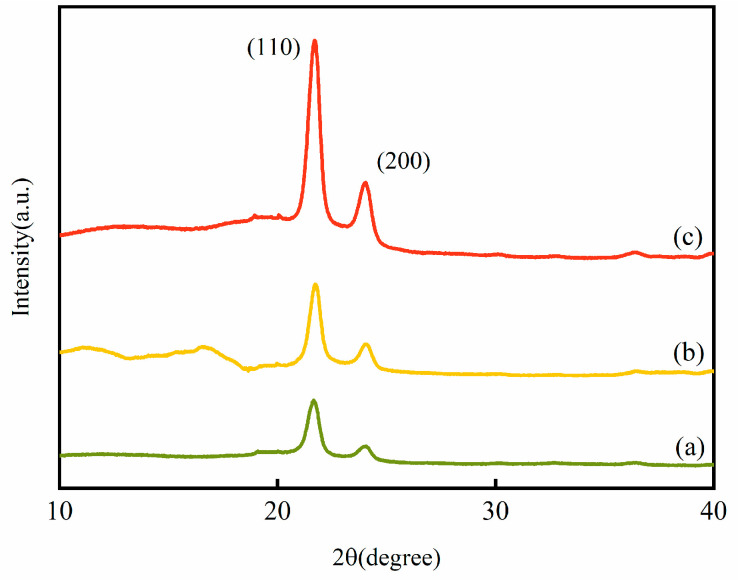
XRD patterns of HDPE/bamboo fiber composites before and after modification: (a) untreated, (b) O_2_ plasma-treated, and (c) N_2_ plasma-treated.

Figure 10 shows the X-ray diffraction of the HDPE/bamboo fiber composites before and after modification.

The 2θ angles of the unmodified composites were about 21° and 24°, respectively, with these being the typical peaks of the (110) and (200) crystallographic planes, respectively, and it can be seen that there are no new diffraction peaks after modification, indicating that the presence of plasma has little effect on the crystal structure of the HDPE/bamboo fiber composites, the peaks at the (110) and (200) crystallographic planes increased, and the peak intensity changed, which was caused by different modification treatments. The treatment of HDPE with N_2_ plasma had more of an effect on the HDPE/bamboo fiber composites than that with O_2_ plasma [42,43].

### 3.10. FTIR Analysis of HDPE/Bamboo Fiber Composites Before and After Modification

**Figure 11:**
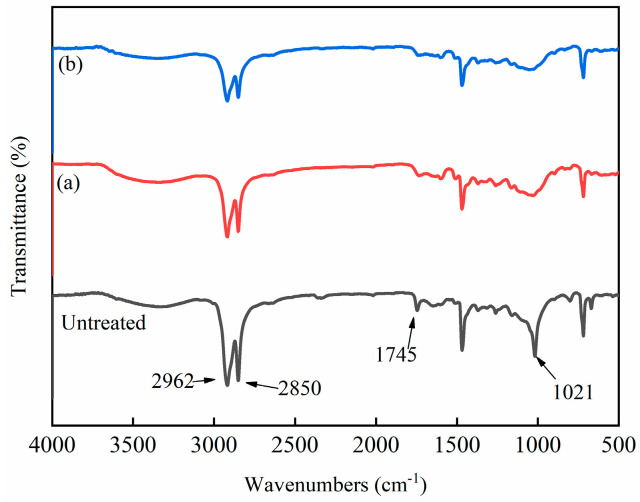
Infrared spectra of HDPE/bamboo fiber composites before and after modification: (a) O_2_ plasma-treated and (b) N_2_ plasma-treated.

Figure 11 shows the FTIR spectra of different modified HDPE/bamboo fiber composites. As can be seen from the figure, the peak at 3420 cm^−1^ belongs to the hydroxyl group because of the physical water absorption that caused O-H stretching, and although the composite was completely dry, it may also have absorbed water from the surrounding environment during the test, and the peak of the hydroxyl group at 3420 cm^−1^ did not change [44]. The peaks at 2850 and 2962 cm^−1^ are due to the expansion and contraction vibrations of saturated hydrocarbons (CH). The peak at 1745 cm^−1^ was generated by the carboxylate group, which was the result of the oxidation reaction during the production of the compound and the high-temperature molding, but it can be seen that the peak decreases significantly in the spectrum after O_2_ and N_2_ plasma treatment, indicating that the plasma treatment had a specific effect on the oxidation reaction of HDPE/bamboo fiber composites, and the peak at 1020 cm^−1^ also weakens to a certain extent [45].

### 3.11. TG Analysis of HDPE/Bamboo Fiber Composites Before and After Modification

**Figure 12:**
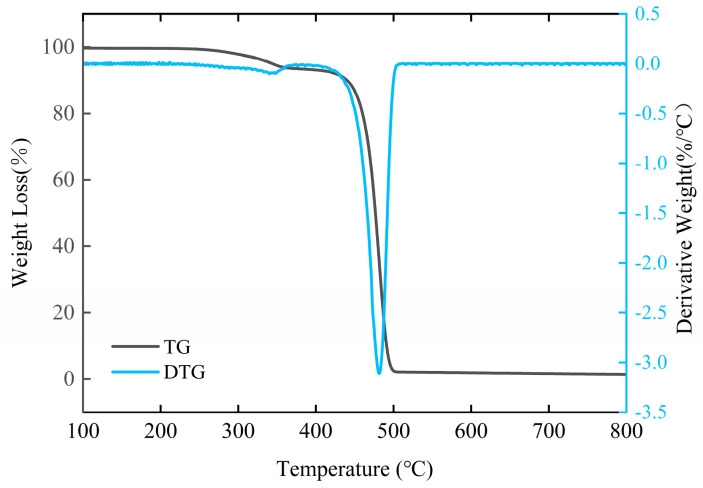
TG/DTG curves of untreated HDPE/bamboo fiber composites.

**Figure 13:**
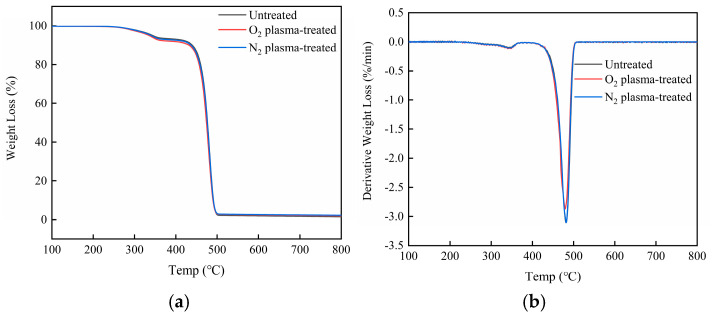
TG/DTG curves of HDPE/bamboo fiber composites: (**a**) TG; (**b**) DTG.

Figure 12 and Figure 13 and Table 8 show the thermal degradation behavior of unmodified and modified HDPE/bamboo fiber composites. Both TG (thermogravimetric) and DTG (derivative thermogravimetric) curves reveal distinct variations after material modification. These differences in thermal degradation patterns are particularly evident in the decomposition temperature shifts and mass loss rates. The observed variation is likely attributable to altered chemical compositions induced by the modification process. From the literature, it is known that the decomposition temperature of hemicellulose and pectin is 250–340 °C, the degradation temperature of cellulose is 340–380 °C, the degradation temperature of lignin is between 380 and 500 °C, and the initial thermal degradation temperature of HDPE is about 430 °C [46,47]. As can be seen in the figure, the thermal degradation of composites occurred in two main stages: the first peak was in the temperature range of 220–370 °C, which corresponded to the degradation of cellulose and hemicellulose, the second peak in the range of 471–550 °C was due to the degradation of HDPE, which also echoes the degradation of lignin in bamboo fiber. The initial degradation temperature T0 of the HDPE/bamboo fiber composites was about 330 ± 30 °C, which was lower than that of HDPE, but higher than that of high-temperature heat-treated bamboo, because the bamboo fibers were wrapped in HDPE, which hindered the degradation of bamboo fibers by the ambient scanning temperature to a certain extent. However, once HDPE reached the degradation point, its degradation rate was very fast, and it can be concluded from the residual weight of the thermogravimetric curve that the residual weight of HDPE was very low, almost zero, and the residual weight of bamboo powder was high [48,49,50]. The interfacial bonding of unmodified composites was weak, because the HDPE in the composites could not completely cover the bamboo fibers, and plasma modification can promote the adhesion between bamboo fibers and the HDPE matrix [51]. After O_2_ plasma modification, the thermal stability of the material was very good, which is consistent with the previous test results.

### 3.12. DSC Analysis of HDPE/Bamboo Fiber Composites Before and After Modification

**Figure 14:**
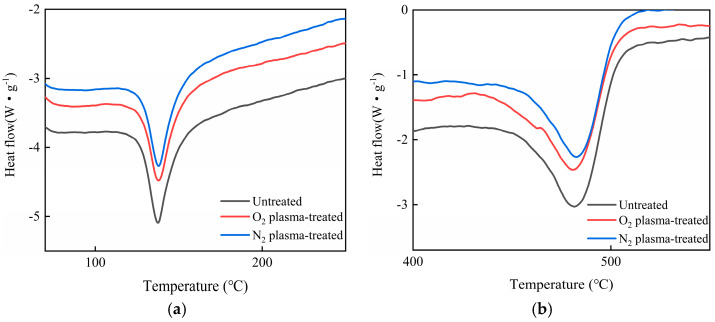
DSC curves of HDPE/bamboo fiber composites: (**a**) O_2_ plasma and (**b**) N_2_ plasma.

DSC analysis is used to determine the possible change in the crystallinity of the matrix after the addition of enhancers and compatibilizers. Corresponding to the crystallization temperature (Tc), crystallization enthalpy (ΔHc), melting temperature (Tm), and melting enthalpy (ΔHm), the degree of crystallinity XC = melting enthalpy/(100% crystallization melting enthalpy) * 100%. In this paper, ΔHC = 270.028 J/g can be calculated for HDPE, as shown in Table 9. Table 9 also shows that the enthalpy of the fusion of the modified HDPE/bamboo fiber composites decreased significantly, while the crystallization enthalpy of the plasma-modified HDPE/bamboo fiber composites increased compared with that of the untreated composite. Figure 14 shows the DSC of different plasma-modified and untreated HDPE/bamboo fiber composites, and it can be seen that the crystallinity and melting temperature of the HDPE/bamboo fiber composites increased with the modification treatment. Combined with the analysis of the aging mechanism of polyethylene, the reason for the characteristic temperature change in the melt peak may be that the molecular chain crosslinking effect mainly occurred in HDPE under the condition of a lower thermal aging temperature, and the molecular chain regularity was improved. The increase in crystallinity ultimately led to improvement in the thermal properties of HDPE, while with the increase in temperature, the breakage of chains in the composites was dominant, the oxidation was intensified, and the influence on melting characteristics was more obvious, with the melting characteristic temperature decreasing and the oxidation stability deteriorating [52,53]. As can be seen in the DSC curves, the changes in the temperature at the start and end points of the melt peak are consistent with those of the short and long chains, which were caused by the breakage of the molecular chains [54,55,56].

## 4. Conclusions

In order to improve the surface wettability of HDPE and further enhance the interfacial compatibility of HDPE/bamboo composite materials, the surface of HDPE was treated with O_2_ or N_2_ plasma in a process optimized using response surface optimization. The effects of plasma treatment on the mechanical properties of HDPE/bamboo were further studied. The surface morphology and surface chemical structure of the materials before and after plasma treatment were analyzed. The conclusions are as follows:

(1) After plasma treatment, the surface energy of HDPE increases significantly and the surface wettability considerably improves because of plasma etching and the introduction of hydrophilic groups.
(2) Optimal processes to improve the surface wettability of HDPE treated with cold O_2_ and N_2_ plasma were determined. The optimal parameters were a treatment power of 1100 W, treatment time of 13 s, and gas flow rate of 1.75 L/min and 2.5 L/min for O_2_ and N_2_, respectively.
(3) Under optimal conditions, the N_2_ plasma treatment yielded a better effect on the wettability of the HDPE surface than O_2_ plasma treatment.
(4) Due to improved interface compatibility, plasma treatment enhances the mechanical properties of HDPE/bamboo fiber composites. After O_2_ plasma treatment, impact strength increased by 19.91% and tensile strength by 16.47%, with corresponding increases of 19.55% in impact strength and 12.48% in tensile strength observed with N_2_ plasma treatment.

## Figures and Tables

**Table 1:** HDPE surface energy after O_2_ plasma treatments.

Sample	Treatment Power (W)	Treatment Time (s)	Gas Flow Rate (L/min)	Contact Angle		Surface Energy (10^−7^ J·cm^−2^)
				H_2_O	CH_2_I_2_	
1	1000	5	2.5	45.6	28.5	56.32
2	1200	5	1.5	47.9	27.62	56.26
3	1200	10	2.5	45.94	25.42	57.36
4	1000	15	2.5	48.38	28.81	54.33
5	1000	10	1.5	45.17	26.69	58.33
6	800	5	1.5	51.11	35.95	50.36
7	800	15	1.5	47.25	28.1	56.51
8	1000	10	1.5	44.16	21.05	57.46
9	1000	5	0.5	52.43	27.6	53.95
10	800	10	2.5	49.1	28.7	55.42
11	1000	10	1.5	46.99	29.78	56.33
12	800	10	0.5	47.27	29	53.95
13	1200	10	0.5	46.52	26.58	57.16
14	1000	10	1.5	44.52	27.24	57.12
15	1000	15	0.5	45.7	25.91	57.73
16	1200	15	1.5	44.86	27.62	57.87
17	1000	10	1.5	44.78	22.91	58.72

**Table 2:** HDPE surface energy after N_2_ plasma treatments.

Sample	Treatment Power (W)	Treatment Time (s)	Gas Flow Rate (L/min)	Contact Angle		Surface Energy (10^−7^ J·cm^−2^)
				H_2_O	CH_2_I_2_	
1	1000	10	1.5	44.96	27.91	57.78
2	1000	15	0.5	51.41	29.84	53.98
3	1000	10	1.5	42.94	28.04	58.83
4	1000	5	0.5	49.56	28.16	55.29
5	1200	10	2.5	30.55	23.96	65.78
6	1000	5	2.5	36.48	23.1	62.97
7	1000	15	2.5	32.21	21.53	65.28
8	800	5	1.5	46.78	35.51	55.32
9	1200	10	0.5	44.78	31.59	57.19
10	800	10	2.5	31.09	22.87	65.66
11	800	15	1.5	41.89	32.08	58.71
12	1000	10	1.5	40.78	29.73	59.61
13	1200	5	1.5	45	36.82	56.09
14	1200	15	1.5	38.05	27.1	61.59
15	800	10	0.5	43.68	33.13	57.52
16	1000	10	1.5	43.01	30.93	58.29
17	1000	10	1.5	41.01	33.93	58.89

**Table 3:** Variance analysis of quadratic model of O_2_ plasma treatment.

Source	Sum of Squares	df	Mean Square	F-Value	*p*-Value	Distinctiveness
Model	59.61	9	6.62	4.85	0.0247	significant
A	19.25	1	19.25	14.09	0.0071	
B	11.42	1	11.42	8.36	0.0233	
C	0.0520	1	0.0520	0.0380	0.8509	
AB	5.16	1	5.16	3.78	0.0931	
AC	0.4018	1	0.4018	0.2941	0.6044	
BC	8.33	1	8.33	6.10	0.0429	
A²	4.00	1	4.00	2.93	0.1307	
B²	7.83	1	7.83	5.73	0.0479	
C²	1.75	1	1.75	1.28	0.2954	
Residual	9.56	7	1.37			
Lack of Fit	5.92	3	1.97	2.17	0.2343	not significant
Pure Error	3.64	4	0.9101			
Cor Total	69.17	16				

**Table 4:** Fit statistics of O_2_ plasma treatment.

Fit Statistics	Value
Std. Dev.	1.17
Mean	56.19
C.V.%	2.08
R^2^	0.8617
Adjusted R^2^	0.6840
Predicted R^2^	−0.4523
Adeq. Precision	7.5518

**Table 5:** ANOVA results of quadratic model for N_2_ plasma treatment.

Source	Sum of Squares	df	Mean Square	F-Value	*p*-Value	Distinctiveness
Model	183.34	9	20.37	22.31	0.0002	significant
A	1.49	1	1.49	1.63	0.2418	
B	34.33	1	34.33	37.59	0.0005	
C	105.49	1	105.49	115.52	<0.0001	
AB	1.11	1	1.11	1.22	0.3065	
AC	0.0517	1	0.0517	0.0566	0.8187	
BC	2.33	1	2.33	2.55	0.1546	
A²	0.0740	1	0.0740	0.0810	0.7842	
B²	1.62	1	1.62	1.77	0.2249	
C²	37.63	1	37.63	41.21	0.0004	
Residual	6.39	7	0.9132			
Lack of Fit	4.49	3	1.50	3.15	0.1483	not significant
Pure Error	1.90	4	0.4752			
Cor Total	189.73	16				

**Table 6:** Fit statistics of N_2_ plasma treatment.

Fit Statistics	Value
Std. Dev.	0.9556
Mean	59.73
C.V.%	1.60
R^2^	0.9663
Adjusted R^2^	0.9230
Predicted R^2^	0.6056
Adeq. Precision	15.5622

**Table 7:** Surface element content of HDPE before and after plasma treatment.

Elements	Untreated	O_2_	N_2_
C	97.69	74.99	80.13
N	0.52	3.01	2.86
O	1.79	22	17
O/C	0.02	0.29	0.21
N/C	0.005	0.04	0.04

**Table 8:** TG weight loss rate.

Sample	Residue (mg)	Weight Loss Rate (%)
Untreated	0.0688	98.54
a	0.1033	98.0248
b	0.1356	97.7755

**Table 9:** DSC analysis parameters.

Sample	T_c0_ (°C)	T_c_ (°C)	T_m0_ (°C)	T_m_ (°C)	ΔH_c_ (J/g)	ΔH_m_ (J/g)	X_c_ (%)
Untreated	124.51	134.41	453.66	484.26	122.37	579.38	21.46
a	124.37	134.51	451.62	484.19	135.34	497.36	18.42
b	124.04	134.62	456.23	458.78	129.81	455.63	16.87

## Data Availability

The original contributions presented in this study are included in the article. Further inquiries can be directed to the corresponding author.

## References

[1] Jinquan X. (2023). Structural and property analysis of high-density polyethylene. China Petoeleum Chem. Stand. Qual..

[2] Li Y. (2023). Preparation, Molding and Properties of Elastomer/Highdensity Polyethylene and Microporous Structure Synergistictoughening Polypropylene. Master’s Thesis.

[3] Wang J., Tian B., Liu J., He J., Zhang S. (2024). Comparative analysis and research on high-density polyethylene production processes. Polyest. Ind..

[4] Cheng X., Wang Y., Wang X., Liu Z., Yang X. (2022). Progress of hydrophilic modification on surface of polymer materials. China Elastomerics.

[5] Yao Y., Liu X., Zhu Y. (1993). Surface modification of high-density polyethylene by plasma treatment. J. Adhes. Sci. Technol..

[6] Su S., Dang X., Shi Z., Sun Z., Zhao H. (2023). Photoelectrochemical detection of ofloxacin based on N_2_ plasma modified thin layer C_3_N_4_/screen-printed electrode. J. Dalian Univ. Technol..

[7] Arpagaus C., Rossi A., Von Rohr P.R. (2005). Short-time plasma surface modification of HDPE powder in a Plasma Downer Reactor–process, wettability improvement and ageing effects. Appl. Surf. Sci..

[8] Mora-Cortes L.F., Rivas-Muñoz A.N., Neira-Velázquez M.G., Contreras-Esquivel J.C., Roger P., Mora-Cura Y.N., So-ria-Arguello G., Bolaina-Lorenzo E.D., Reyna-Martínez R., Zugasti-Cruza A. (2022). Biocompatible enhancement of poly(ethyleneterephthalate) (PET) wastefilms by coldplasma aminolysis. J. Chem. Technol. Biotechnol..

[9] Yoshihisa K., Yoshimura A., Shibamori Y., Fuchigami K., Kubota N. (2013). Hydrophilic modification of plastic surface by using microwave plasma irradiation. IHI Eng. Rev..

[10] Zuo W. (2023). Advances in the Study of Droplet lmpact Behaviour on the Surface of Superhydrophobic Structures. Mater. Res. Appl..

[11] Hong W., Guan D., Guo H., Bin Y. (2013). Influence of dielectric barrier discharge (DBD) cold plasma treatment on wettability of Pinus yunnanensis wood. China For. Sci. Technol..

[12] (2018). Plastics-Determination of Charpy Impact Properties-Part 2: Instrumented Impact Test.

[13] (2008). Plastics-Determination of Flexural Properties.

[14] (2006). Plastics-Determination of Tensile Properties.

[15] Ya C., Wen L., Ya Y. (2023). Optimization of Laser Paint Removal Process for Carbon Fiber Composite Substrate Based on Response Surface Analysis. Chin. J. Lasers.

[16] Bin L., Yong Z., Rui L., Peng S., Ting Z., Xue Y., Bo L. (2019). Optimization of preparation process of birch veneer/glass fiber composite. J. Beijing For. Univ..

[17] Zeng D., Li C., Li Y., Zhang X., Huang Q. (2018). Optimization of Polyvinyl Alcohol Chitosan Hydrogel Formulation by Response Surface Analysis. Guangdong Chem. Ind..

[18] Shi L., Zhang D., Hu M., Wen M., Li Z., Qin H. (2018). Optimization of Preparations of MIP-N-TiO2 by Response Surface Methodology. Guangzhou Chem. Ind..

[19] He D., Pan Z., Wang H., Du W. (2022). Optimizing the content of activated crumb rubber and SBR composite modified asphalt by response surface methodology-grey relation analysis. New Chem. Mater..

[20] Yu X., Li Y., Xu S. (2024). Optimization of Extraction Process of Banana Pseudostem Fibers and Its Characterization. Biomass Chem. Eng..

[21] Chen S., Zhang J., Jiang H. (2019). Application of response surface methodologyin optimizing flocculation-coagulation process of sweet potato wastewater. Food Ferment. Ind..

[22] Li L., Chai H., Wei Q., Xue J. (2024). Optimizing the extraction craft of polysaccharides from Blumea balsamifera by ultrasonic method based on response surface modeling. Chem. Eng..

[23] Yu S. (2014). Preparation and Performance of PET Fibers and Bamboo Fibers Reinforced Unsaturated Polyester Hybrid Composites. Master’s Thesis.

[24] Lihua T. (2008). Surface Modification of PolymericMaterials by Cold Plasma Treatment. Master’s Thesis.

[25] Wang H., Du G., Zheng R., Wang H., Li Q. (2014). Bonding Performance of Wood Treatment by Oxygen and Nitrogen Cold Plasma. Agric. Sci. Technol..

[26] Ghobeira R., Tabaei P.S.E., Morent R., Geyter N.D. (2022). Chemical characterization of plasma-activated polymeric surfaces via XPS analyses: A review. Surf. Interfaces.

[27] Zeng S.F., Guo P., Hu C.Y., Zhi W. (2023). Effects of mechanical recycling on optical properties and microstructure of recycled high-density polyethyle-ne pellets and bottles. J. Appl. Poly-Mer Sci..

[28] Chen Y., Ma H., Sun M., Yang S., Chen J., Zhou X. (2023). Research on cold plasma modified PLA fiber/TPS composites. Compos. Sci. Eng..

[29] Wu S., Chen Z., Xu X. (2003). Study on structure and properties of HDPE functionalized by ultravi-olet irradiation in air and O2 atmosphere. Mat-Erials Lett..

[30] Nicole M.S., Laurent M.M. (2004). Surface chemistry changes of weathered HDPE/wood-flour composites studied by XPS and FTIR spectr-oscopy. Polym. Degrad. Stab..

[31] Ghanadi M., Padhye L.P. (2024). Revealing the Long-Term Impact of Photodegradation and Fragmentation on HDPE in the Marine Environment: Origins of Microplastics and Dissolved Organics. J. Hazard. Mater..

[32] Xie L., Li S. (2010). Surface Modification of High Density Polyethylene Film by Low Temperature O_2_ Plasma Treatment. Polym. Mater. Sci. Eng..

[33] Bhowmik S., Ghosh P.K., Ray S., Barthwal S.K. (1998). Surface modification of high density polyethylene and polypropylene by DC glow discharge and adhesive bonding to steel. J. Adhes. Sci. Technol..

[34] Zhao Y. (2023). Practice of Applying X-ray Photoelectron Spectrometer to Experimental Teaching of Undergraduates. Res. Explor. Lab..

[35] Rashid B., Leman Z., Jawaid M., Ghazali M.J., Ishak M.R., Abdelgnei M.A. (2017). Dry sliding wear behavior of untreated and treated sugar palmfiber filled phenolic composites using factorial technique. Wear.

[36] Swain P.T.R., Biswas S. (2017). Abrasive wear behaviour of surface modified jute fiber reinforced epoxycomposites. Mater. Res..

[37] Ru W. (2011). Preparation and Performance Research for Wood Flour Filled Polypropylene. Master’s Thesis.

[38] Yu F., Song J., Wu Q., Chen L., Yang W. (2013). Bamboo flour modifying by grafted improves mechanical property of bamboo-plastic composites. Trans. Chin. Soc. Agric. Eng..

[39] Yang Z. (2017). Reasearch on the Structure Design and Properties of Bamboo Plastic Composite. Ph.D. Thesis.

[40] Niu Y., Du X., Yu M., Hong W., Gao S. (2024). Preparation of nano-SiO_2_@TiO_2_ reinforced bamboo-plasticcomposites and simulation analysis of performance for pallet application. Trans. Chin. Soc. Agric. Eng..

[41] Guo Y., Zhu S., Chen Y., Li D. (2019). Thermal properties of wood-plastic composites with different compositions. Materials.

[42] Chen J., Yan N. (2013). Crystallization behavior of organo-nanoclay treated and untreated kraft fiber–HDPE composites. Compos. Part B Eng..

[43] Xian Y., Li H., Wang C., Wang G., Ren W., Cheng H. (2015). Effect of white mud as a second filler on the mechanical and thermal properties of bamboo residue fiber/polyethylene composites. BioResources.

[44] Shieh Y.-T., Liu C.-M. (1999). Silane grafting reactions of LDPE, HDPE, and LLDPE. J. Appl. Polym. Sci..

[45] Song W., Zhang S., Fei B., Zhao R. (2021). Effect of monomer type on polydopamine modification of bamboo flour and the resulting interfacial properties of bamboo plastic composites. Ind. Crops Prod..

[46] Cheng S., Lau K., Liu T., Yong Z., Pou-Man L., Yan Y. (2009). Mechanical and thermal properties of chicken feather fiber/PLA green composites. Compos. Part B Eng..

[47] Ahmad E.E.M., Luyt A.S. (2012). Effects of organic peroxide and polymer chain structure on morphology and thermal properties of sisal fibre reinforced polyethylene composites. Compos. Part A Appl. Sci. Manuf..

[48] Shah A.A., Hasan F., Hameed A., Ahmed S. (2008). Biological degradation of plastics: A comprehensive review. Biotechnol. Adv..

[49] Torki A.M., Stojanović D.B., Živković I.D., Marinković A., Škapin S.D., Uskoković P.S., Aleksić R.R. (2012). The viscoelastic properties of modified thermoplastic impregnated multiaxial aramid fabrics. Polym. Compos..

[50] Lan D. (2014). Mechanical and Thermal Properties of Bamboo and High Density Polyethylene (HDPE) Composites with Heat-treated Bamboo Flour. Master’s Thesis.

[51] Guo Y., Wang L., Wang H., Chen Y., Zhu S., Chen T., Luo P. (2020). Properties of bamboo flour/high-density polyethylene composites reinforced with ultrahigh molecular weight polyethylene. J. Appl. Polym. Sci..

[52] Shu S., Fei Z., Yu W., Jun Y., Qiang Y., Meng W. (2021). Application of a Differential Scanning Calorimeter in the Inspection and Control of Wood-Plastic Raw Materials. Plast. Addit..

[53] Jiao D. (2013). Studies on Component Analysis and Chlorine WaterResistance of Polyolefin/Wood Flour Composites. Master’s Thesis.

[54] Jun D., Hua Y., Junjun G., De H., Jian Y. (2016). Study on Degradation Properties of High Density Polyethylene by DSC In-situ Accelerated Thermal Aging. Mater. Rep..

[55] Menard K., Menard N. (2017). Thermal analysis of polyethylene. Handbook of Industrial Polyethylene and Technology: Definitive Guide to Manufacturing, Properties, Processing, Applications and Markets.

[56] Sirisinha K., Boonkongkaew M., Kositchaiyong S. (2010). The effect of silane carriers on silane grafting of high-density polyethylene and properties of crosslinked products. Polym. Test..

